# A Phase 1 Study of ABI‐009 (*Nab*‐sirolimus) in Combination With Temozolomide and Irinotecan in Pediatric Patients With Recurrent or Refractory Solid Tumors, Including CNS Tumors—A Children's Oncology Group Pediatric Early Phase Clinical Trial Network Study ADVL1514

**DOI:** 10.1002/cam4.70376

**Published:** 2024-11-02

**Authors:** Stuart L. Cramer, Alyssa Terry Reddy, Charles Gene Minard, Stephan Voss, Elizabeth Fox, Xiaowei Liu, Kristina Denic, Joel M. Reid, Brenda J. Weigel

**Affiliations:** ^1^ Prisma Health‐Midlands Children's Center for Cancer and Blood Disorders Columbia South Carolina USA; ^2^ Neurology/Child Neurology UCSF Medical Center‐Mission Bay San Francisco California USA; ^3^ Baylor College of Medicine/Dan L Duncan Comprehensive Cancer Center Dan L. Duncan Institute Houston Texas USA; ^4^ Dana Farber Cancer Institute Children's Hospital of Boston Boston Massachusetts USA; ^5^ Cancer Center, Clinical Trials Administration Saint Jude Children's Research Hospital Memphis Tennessee USA; ^6^ Children's Oncology Group Monrovia California USA; ^7^ Mayo Clinic Rochester Minnesota USA; ^8^ Department of Pediatrics, Hem/Onc/BMT University of Minnesota Medical Center‐Fairview Minneapolis Minnesota USA

**Keywords:** ABI‐009, Clinical trial, *nab*‐rapamycin, Pediatrics, rapamycin

## Abstract

**Background:**

*Nab*‐sirolimus (ABI‐009, *nab*‐rapamycin; Aadi Bioscience Inc. [Aadi]) is a human albumin‐bound form of sirolimus nanoparticles, a potent mTOR inhibitor. This phase I trial was conducted to define dose‐limiting toxicities (DLT), maximum tolerated or recommended phase II dose (MTD/RP2D), and pharmacokinetics of *Nab*‐sirolimus in combination with temozolomide and irinotecan.

**Methods:**

Using a rolling 6 design, *Nab*‐sirolimus was administered intravenously (IV) on days (D) 1 and 8 of cycle (C) 1. In subsequent cycles, *Nab*‐sirolimus was administered D1 and D8 in combination with temozolomide (125 mg/m^2^/dose, maximum 250 mg/dose) and irinotecan (90 mg/m^2^/dose) orally, daily on D1–5. Cycle duration was 21 days. Three dose levels (DL) of *Nab*‐sirolimus were investigated (DL1: 35 mg/m^2^/dose, DL‐1: 20 mg/m^2^/dose, and DL‐2: 15 mg/m^2^/dose). The observation period for estimating the MTD/RP2D was defined by cycles 1 and 2.

**Results:**

Thirty‐three patients were enrolled, 32 were eligible. Dose determination included 17 evaluable patients, median (range) age 12 (2–20) years and six additional patients were enrolled (four evaluable for toxicity) on a pharmacokinetic cohort. C1 or C2 DLTs were primarily thrombocytopenia including 2/5 patients at DL1, 2/6 patients at DL‐1, and 1/6 patients at DL‐2. One patient (DL1) with Ewing Sarcoma had a partial response and remained on study for 35 cycles. Rapamycin clearance was dose dependent. Irinotecan clearance and its active metabolite SN‐38 exposure were not affected by coadministration with *Nab*‐sirolimus.

**Conclusion:**

The MTD for *Nab*‐sirolimus was 15 mg/m^2^/dose IV on D1 and D8 in combination with temozolomide 125 mg/m^2^/dose and oral irinotecan 90 mg/m^2^/dose daily for 5 days during 21D cycles.

**Trial Registration:**

ClinicalTrials.gov identifier NCT02975882

## 1 Introduction

There are few effective treatment options for children with relapsed or refractory solid and CNS tumors. *Nab*‐sirolimus (also known as ABI‐009, *nab*‐rapamycin) is a human albumin‐bound nanoparticle injectable form of rapamycin, a potent allosteric inhibitor of mTORC1. Rapamycin interacts with the 12 kDa FK506‐binding protein and forms a gain‐of‐function complex, resulting in inhibition of mTOR function [1]. Aberration of the mTOR pathway occurs in numerous pediatric cancers as a result of gain‐of‐function mutations in oncogenes and/or loss‐of‐function mutations in tumor suppressors, which results in tumor growth, angiogenesis, evading cell death, and metastasis [1, 2, 3, 4, 5, 6, 7].

*Nab*‐sirolimus was developed using albumin nanoparticle technology [8]. This enhances the accumulation of albumin‐bound drug in solid tumors, and may improve drug delivery, efficacy, and reduce the risk of anaphylaxis [8, 9].

There have been numerous clinical trials evaluating mTOR inhibition in combination with a variety of chemotherapy backbone regimens in both adults and children. The Children's Oncology Group (COG) has investigated mTOR inhibition in combination with cyclophosphamide and vinorelbine (ARST0921) and in combination with irinotecan and temozolomide (ADVL0918) [2, 10]. The COG Phase 1 trial (ADVL0918) evaluated temsirolimus in combination with irinotecan and temozolomide and identified the recommended phase 2 dose (RP2D) as temsirolimus (35 mg/m^2^) on days 1, 8, and 15 with oral irinotecan (90 mg/m^2^) and temozolomide (125 mg/m^2^) on days 1–5 of a 21‐day cycle [2].

For this trial, the *Nab*‐sirolimus starting dose (35 mg/m^2^/dose) was based on pharmacokinetics (PK) of temsirolimus [11] and *Nab*‐sirolimus [9] in adults. Temsirolimus is rapidly hydrolyzed to the active metabolite sirolimus. Temsirolimus (45 mg/m^2^) yielded a total exposure (area under concentration × time curve, AUC) of 15,154 ng·h/mL (temsirolimus 3414 ng·h/mL, sirolimus 11,740 ng·h/mL). *Nab*‐sirolimus (45 mg/m^2^) yielded a sirolimus AUC of 24,564 ng·h/mL, exceeding the total exposure of temsirolimus (45 mg/m^2^). In ADVL0918, temsirolimus (35 mg/m^2^) days 1,8, and 15 was well tolerated in combination with irinotecan and temozolomide. Therefore, the *Nab*‐sirolimus starting dose on our trial was 35 mg/m^2^ Day 1 and 8. We expected the sirolimus AUC after *Nab*‐sirolimus to exceed the sirolimus AUC after temsirolimus.

ADVL1514 was a multi‐institutional dose escalation study of *Nab*‐sirolimus in combination with temozolomide and irinotecan in children with recurrent or refractory solid and CNS tumors. The primary objectives were to estimate the maximally tolerated dose (MTD)/RP2D of *Nab*‐sirolimus administered as an intravenous infusion on days 1 and 8 as a single agent during cycle 1, and in combination with temozolomide and irinotecan (days 1–5) in cycle 2; and characterize *Nab*‐sirolimus and irinotecan PK. Secondary aim was to define the antitumor activity of *Nab*‐sirolimus in combination with temozolomide and irinotecan within the confines of a Phase 1 study.

## 2 Patients and Methods

### 2.1 Patient Eligibility

Patients ≥ 12 months and ≤ 21 years of age with recurrent or refractory solid tumors, including CNS tumors refractory to standard treatment or for whom no known curative therapy existed were eligible. Adequate organ function including absolute neutrophil count (ANC) > 1000/mm^3^ and platelet count > 100,000/mm^3^ (transfusion independent) were required. Patients were excluded if they had previously received irinotecan and temozolomide in combination and had significant toxicity or progression of disease, prior combination therapy with irinotecan, temozolomide, and an mTOR inhibitor, BSA of ≤ 0.2 m^2^, known bone marrow metastasis, interstitial lung disease or pneumonitis, current deep vein thrombosis or deep vein thrombosis within the past 6 months, or history of allergic reaction to albumin.

The protocol was approved by the National Cancer Institute (NCI) Cancer Therapeutics Evaluation Program and the NCI pediatric central institutional review board. The study was conducted in accordance with the principles of the World Medical Association Declaration of Helsinki. Informed consent and child assent, when appropriate, were obtained from all participants and/or parents or legal guardians.

### 2.2 Study Design and Administration of Protocol Therapy

*Nab*‐sirolimus was supplied and distributed by Aadi, and administered as a single agent intravenously on days 1 and 8 of the initial 21‐day cycle. For subsequent cycles, *Nab*‐sirolimus was administered on days 1 and 8 in combination with oral temozolomide and irinotecan, on days 1–5 of each cycle. Cefixime prophylaxis for irinotecan‐associated diarrhea was required. Protocol therapy could continue until patients experienced disease progression, met discontinuation of protocol therapy criteria, or received a maximum of 35 cycles (24 months).

A rolling 6 design was used to escalate *Nab*‐sirolimus (starting dose DL1 35 mg/m^2^/dose); irinotecan and temozolomide doses were constant (temozolomide 125 mg/m^2^/dose [maximum 250 mg/dose] orally, and irinotecan 90 mg/m^2^/dose orally), daily on days 1–5 of 21‐day cycles. Initially, *Nab*‐sirolimus dose escalations to DL2 (45 mg/m^2^/dose) and DL3 (55 mg/m^2^/dose) were planned; however, due to dose‐limiting thrombocytopenia related to *Nab*‐sirolimus monotherapy in cycle 1, dose escalation was not feasible. The trial was amended to include DL‐1 (20 mg/m^2^/dose) and DL‐2 (15 mg/m^2^/dose) and modify the monitoring and definitions of dose‐limiting thrombocytopenia. The MTD was defined as the dose level in which fewer than one third of patients experienced a dose‐limiting toxicity (DLT) in cycles 1 and 2. Once the MTD/RP2D was defined, up to six additional patients were enrolled to acquire toxicity and PK data in at least six patients ≤ 12 years old.

Toxicities were graded according to the NCI Common Terminology Criteria for Adverse Events (CTCAE v 5). The DLT observation period for dose escalation and determination of MTD/RP2D was the combination of both first and second cycle of protocol therapy and included toxicities that were at least possibly attributed to *Nab*‐sirolimus alone or in combination with irinotecan and temozolomide. Patients who experienced a DLT during cycle 1 or 2 and patients without DLT who received at least 85% of the prescribed protocol therapy in both cycles 1 and 2 and completed toxicity monitoring, were evaluable for determination of the MTD.

Initially, hematological DLT was defined as grade 4 neutropenia (ANC < 500/mm^3^) or grade 2 thrombocytopenia (platelets< 75,000/mm^3^) on Day 8 that did not resolve to ANC ≥ 750/mm^3^ and platelets ≥ 75,000/mm^3^ (transfusion independent) by Day 11, grade 4 thrombocytopenia (platelets < 25,000/mm^3^) or grade 4 anemia not due to malignant infiltration, grade 4 neutropenia for > 7 days duration, grade 3 thrombocytopenia with clinically significant bleeding, petechiae or purpura, or that persists for ≥ 7 days, and/or that required platelet transfusion. After patients on DL 1 and DL‐1 had been evaluated, an amendment added DL‐2 and modified hematological DLT to grade 4 thrombocytopenia (< 50,000/mm^3^) on Day 8 that did not resolve to platelets ≥ 50,000/mm^3^ (transfusion independent) by Day 11.

Non‐hematological DLT was defined as grade ≥ 3 toxicity possibly, probably, or definitely attributed to protocol therapy with the following exceptions: grade 3 nausea and vomiting < 3 days duration; grade 3 liver enzyme elevation (ALT/AST/GGT) that returned to grade ≤ 1 or baseline prior to the next treatment cycle; grade 3 fever, grade 3 infection, grade 3 hypophosphatemia, hypokalemia, hypocalcemia, or hypomagnesemia responsive to supplementation; grade 3 or 4 hypertriglyceridemia that returned to grade ≤ 2 prior to the next treatment cycle; grade 3 hyperglycemia that returned to a fasting glucose value ≤ 250 mg/dL or a fasting glucose of value ≤ 13.9 mmol/L prior to the next treatment cycle; and grade 3 or 4 hypercholesterolemia that returned to grade ≤ 2 after initiation of lipid lowering medication prior to the next treatment cycle.

Hematological or non‐hematological toxicity that resulted in a > 21‐day delay in the planned initiation of a subsequent cycle of therapy was considered a DLT and patients were removed from protocol therapy.

Dose modification for toxicity was a reduction in the *Nab*‐sirolimus dose level; no reduction in temozolomide and irinotecan doses were planned because these doses are known to provide benefit and are standard of care. If more than two dose reductions of *Nab*‐sirolimus were required, the patient was removed from protocol therapy.

### 2.3 Disease Response Evaluations

Disease evaluations were obtained at baseline, the end of the first and second cycle, every other cycle x 2 and then every three cycles. Disease response for solid tumors was assessed according to the revised Response Evaluation Criteria in Solid Tumors (RECIST v1.1) and neuroblastoma was assessed using bone marrow biopsies, anatomic imaging for measurable disease, and MIBG scintigraphy for evaluable MIBG‐avid tumors. Central imaging review was completed for all participants who had institutional assigned objective response or had stable disease for six or more cycles. Best overall response was reported for each patient who had at least one radiographic assessment after initiation of protocol therapy or clinically progressive disease. Patients experiencing progressive disease in cycle 1 could continue to cycle 2 if they did not experience a DLT in cycle 1 and they did not meet any protocol defined off study criteria. Patients were required to have no evidence of clinical progression and at least stable disease by RECIST v 1.1 (when imaging was required) to continue to cycle 3 and beyond.

### 2.4 Pharmacokinetic Analysis

#### 2.4.1 Rapamycin

Blood samples (2 mL in potassium K2EDTA) for rapamycin PK analysis were collected on Day 1, cycle 1, and cycle 2 before the 30‐min *Nab*‐sirolimus infusion and 30 min, 1, 2, 4, 8, 24, and 72 h after the beginning of infusion. An additional sample was collected immediately prior to the Day 8 *Nab*‐sirolimus infusion. The rapamycin assay was performed by Aadi using a validated method for the quantitation. The method utilized liquid extraction followed by LC–MS/MS with rapamycin‐13CD3 as an internal standard. The analytical range was 1–400 ng/mL for rapamycin. Analysis was conducted in compliance with applicable AIT Bioscience Operating Procedures.

#### 2.4.2 Irinotecan

Blood samples (sodium heparin) for irinotecan PK analysis were collected on cycle 2 Day 1 before the irinotecan oral dose and 10 min, 1, 3, 6, and 24 h after the irinotecan dose. After collection, tubes were gently mixed, immediately centrifuged (10 min, 2500*g*, 5°C), and plasma was transferred into two separate plastic tubes. Plasma samples were assayed for irinotecan, and its metabolites SN‐38, and APC (7‐ethyl‐10‐[4‐N‐(5‐aminopentanoic acid)‐1‐piperidino]carbonyloxy‐camptothecin) using validated, sensitive, and specific isocratic high‐performance liquid chromatography (HPLC) methods [12].

Blood (rapamycin) and plasma (irinotecan) concentration–time data were analyzed via non‐compartmental analysis using Phoenix WINNONLIN (Certara). The apparent terminal elimination rate constants (λz) were determined via least‐squares regression of the log‐transformed plasma concentration versus time data for the last 3–4 time points. The apparent elimination half‐life (t1/2) was calculated as 0.693/λz. The area under the blood (rapamycin) or plasma (irinotecan) concentration–time curves (AUC_0‐T_) were determined using the linear trapezoidal rule from time zero to 192 h (rapamycin) or 24 h (irinotecan). The area under the plasma concentration–time curves through infinite time (AUC_inf_) values for rapamycin and irinotecan were calculated by adding CT/λ_z,rapamycin_ to AUC_0‐192h,rapamycin_ or CT/λ_z,irinotecan_ to AUC_0‐24h,irinotecan_. The clearance (CL) of rapamycin and irinotecan were calculated as dose/AUC_inf_. The administered dose of irinotecan (CPT‐11) was expressed in free base equivalents. Descriptive statistics were used to summarize PK parameters.

## 3 Results

Between July 2017 and June 2021, 33 patients enrolled; one was ineligible due to absence of measurable or evaluable disease at baseline. For dose determination, 17 patients were evaluable for toxicity and determination of the MTD/RP2D; nine were not evaluable because they did not receive the required 85% of protocol therapy during cycles 1 and 2 and were removed from protocol therapy either due to disease progression or physician discretion. In addition, 4/6 patients enrolled to the PK cohort were evaluable for toxicity. Patient characteristics are presented in Table 1.

**TABLE 1:** Patient characteristics.

	Toxicity evaluability		
	No (*N* = 11)	Yes (*N* = 21)	Total (*N* = 32)
Age			
*N*	11	21	32
Median	12.0	12.0	12.0
Range	1.0, 21.0	2.0, 20.0	1.0, 21.0
Gender, *n* (%)			
Male	10 (90.9%)	12 (57.1%)	22 (68.8%)
Female	1 (9.1%)	9 (42.9%)	10 (31.3%)
Race, *n* (%)			
White	5 (45.5%)	13 (61.9%)	18 (56.3%)
Asian	2 (18.2%)	0 (0.0%)	2 (6.3%)
Black or African American	1 (9.1%)	4 (19.0%)	5 (15.6%)
Multiple races	1 (9.1%)	1 (4.8%)	2 (6.3%)
Unknown	2 (18.2%)	3 (14.3%)	5 (15.6%)
Ethnicity, *n* (%)			
Hispanic or Latino	2 (18.2%)	2 (9.5%)	4 (12.5%)
Not hispanic or latino	9 (81.8%)	18 (85.7%)	27 (84.4%)
Unknown	0 (0.0%)	1 (4.8%)	1 (3.1%)
Prior chemotherapy			
*N*	11 (100%)	21 (100%)	32 (100%)
Median	3.0	2.0	2.0
Range	1.0, 7.0	1.0, 6.0	1.0, 7.0
Prior radiation			
*N*	9 (82%)	17 (81%)	26 (81%)
Median	1.0	1.0	1.0
Range	1.0, 2.0	1.0, 2.0	1.0, 2.0
Diagnosis disease, *n* (%)			
Alveolar rhabdomyosarcoma	1 (9.1%)	1 (4.8%)	2 (6.3%)
Astrocytoma, NOS	0 (0.0%)	1 (4.8%)	1 (3.1%)
Choroid plexus carcinoma	1 (9.1%)	0 (0.0%)	1 (3.1%)
Desmoplastic small round cell tumor	1 (9.1%)	0 (0.0%)	1 (3.1%)
Ependymoma, NOS	1 (9.1%)	0 (0.0%)	1 (3.1%)
Ependymoma, anaplastic	1 (9.1%)	0 (0.0%)	1 (3.1%)
Ewing sarcoma	1 (9.1%)	4 (19.0%)	5 (15.6%)
Hepatoblastoma	1 (9.1%)	1 (4.8%)	2 (6.3%)
Malignant rhabdoid tumor	1 (9.1%)	1 (4.8%)	2 (6.3%)
Medulloblastoma, NOS	1 (9.1%)	1 (4.8%)	2 (6.3%)
Nephroblastoma, NOS	0 (0.0%)	3 (14.3%)	3 (9.4%)
Neuroblastoma, NOS	0 (0.0%)	2 (9.5%)	2 (6.3%)
Osteosarcoma, NOS	1 (9.1%)	3 (14.3%)	4 (12.5%)
Pancreatoblastoma	0 (0.0%)	1 (4.8%)	1 (3.1%)
Pineoblastoma	0 (0.0%)	1 (4.8%)	1 (3.1%)
Small cell osteosarcoma	0 (0.0%)	1 (4.8%)	1 (3.1%)
Undifferentiated sarcoma	1 (9.1%)	1 (4.8%)	2 (6.3%)

During dose escalation, thrombocytopenia was the most frequent DLT and occurred in cycle 1 (*Nab*‐sirolimus alone) and cycle 2 (*Nab*‐sirolimus in combination with irinotecan and temozolomide) (Table 2). At DL1 (35 mg/m^2^), 2/5 patients experienced dose‐limiting thrombocytopenia during cycle 1 (*n* = 1) or cycle 2 (*n* = 1); at DL‐1, 2/6 patients experienced dose‐limiting thrombocytopenia in cycle 1; at DL‐2, 1/6 patients experienced dose‐limiting thrombocytopenia in cycle 1. One of four patients evaluable for toxicity in the PK cohort experienced a non‐hematological DLT (oral mucositis). Overall, at DL‐2 (15 mg/m^2^), there were two DLTs among 10 evaluable patients. Thus, the MTD for *Nab*‐sirolimus was 15 mg/m^2^/dose days 1 and 8 in cycle 1 and in combination with five daily doses of temozolomide 125 mg/m^2^/dose and oral irinotecan 90 mg/m^2^/dose in cycle 2.

**TABLE 2:** Summary of dose‐limiting toxicities.

Dose level (DL)	*Nab*‐sirolimus day 1 and 8 cycle 1	Irinotecan (IRN) and temozolomide (TMZ) orally, daily days 1–5 starting cycle 2	No. patients evaluable	No. patients cycle 1 DLTs	No. patients cycle 2 DLTs
DL1	35 mg/m^2^/dose	IRN 90 mg/m^2^/dose;	5	1	1
Dose escalation cohort		TMZ 125 mg/m^2^/dose		(Thrombocytopenia)	(Thrombocytopenia)
		(max 250 mg)			
DL −1	20 mg/m^2^/dose	IRN 90 mg/m^2^/dose	6	2	
Dose escalation cohort		TMZ 125 mg/m^2^/dose		(Thrombocytopenia)	
		(max 250 mg)			
DL‐2	15 mg/m^2^/dose	IRN 90 mg/m^2^/dose	6	1	
Dose escalation cohort		TMZ 125 mg/m^2^/dose		(Thrombocytopenia)	
		(max 250 mg)			
DL −2	15 mg/m^2^/dose	IRN 90 mg/m^2^/dose	4	1	
PK		TMZ 125 mg/m^2^/dose		(Mucositis)	
		(max 250 mg)			

The median (range) number of cycles administered to eligible patients was 1.5 (1–35) and to patients evaluable for toxicity was 3 (1–35). In 21 participants evaluable for toxicity, frequent (≥ 10%) non‐dose‐limiting Grade ≥ 3 adverse events at least possibly related to protocol therapy, included anemia, decreased WBC, decreased ANC, decreased lymphocyte count, increased ALT, and hypophosphatemia (Table S1).

### 3.1 Pharmacokinetics

**FIGURE 1:**
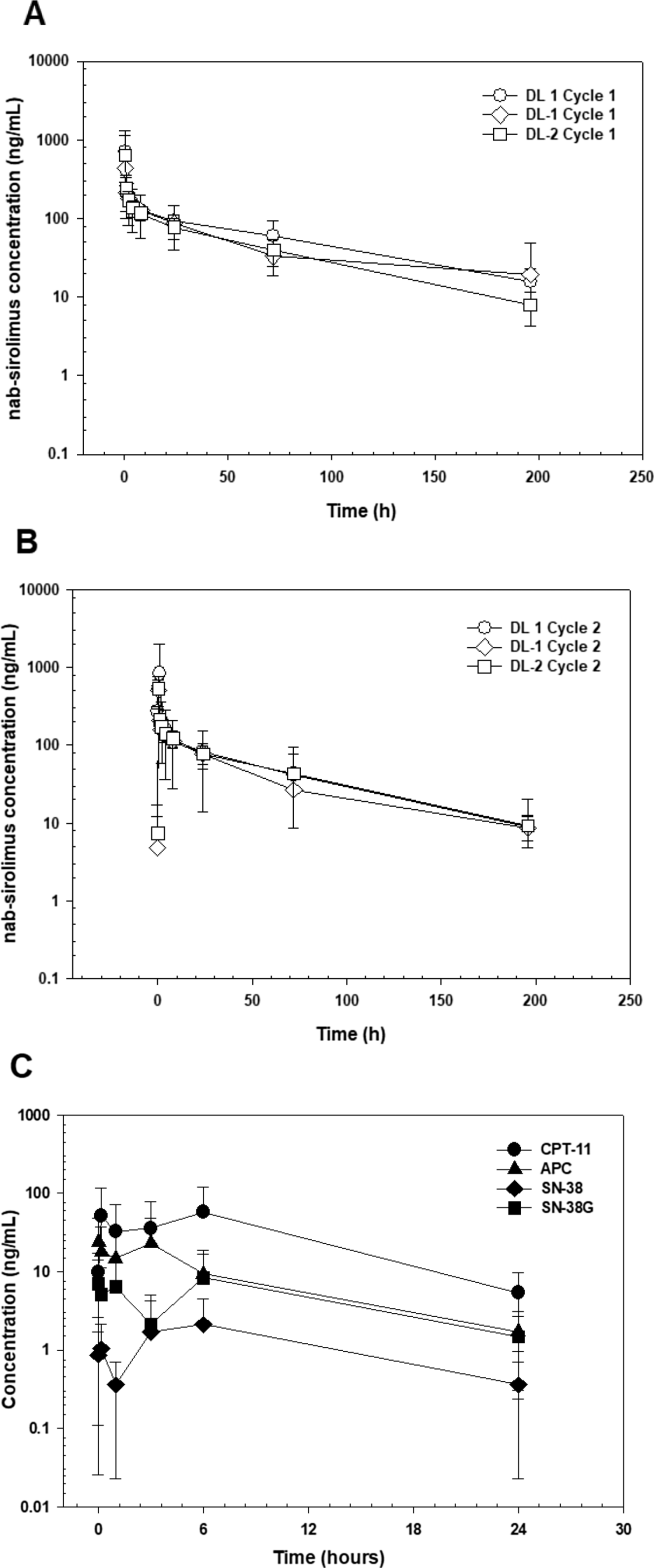
Graphs of *Nab*‐sirolimus blood concentration versus time in cycle 1 (A) and cycle 2 (B) for each dose level. Graph of the plasma concentration versus time for irinotecan and its metabolites in cycle 2 Day 1 (C).

A total of 31 patients in cycle 1 and 15 patients in cycle 2 were evaluable for the PK characterization of rapamycin (Table 3). Concentration versus time data for cycle 1 and cycle 2 are shown in Figure 1A,B, respectively. PK of rapamycin did not appear to be dose proportional. In cycle 1, the rapamycin *C*_max_ and AUC_0‐inf_ decreased by 41% and 34%, respectively, as the *Nab*‐sirolimus dose decreased 60% from 35 mg/m^2^ to 15 mg/m^2^. Consistent with the lower‐than‐expected change in *C*_max_ and AUC_0‐inf_, the CL_B_ decreased 34% over the same dose range. When administered with irinotecan in cycle 2, rapamycin *C*_max_ and AUC_0‐inf_ decreased by 23% and 47%, respectively, while CL_B_ decreased 33% (Table 3). The difference in clearance in patients receiving 15 and 35 mg/m^2^ dose levels was statistically significant for cycle 1 (*p* = 0.0268), but not for cycle 2 (*p* = 0.200) (Figure S2). The BSA‐adjusted CL_B_ appeared to be higher for patients < 12 years compared with those ≥ 12 years of age in both cycle 1 (2.63 ± 0.85, *n* = 15, vs. 1.93 ± 0.54, *n* = 16) and cycle 2 (2.54 ± 1.02, *n* = 9, vs. 1.72 ± 0.26, *n* = 7). The BSA‐adjusted CL_B_ appeared to be lower for males compared to females in cycle 1 (2.10 ± 0.54, *n* = 21, vs. 2.63 ± 0.91, *n* = 10) and in cycle 2 (1.80 ± 0.40, *n* = 9 vs. 2.47 ± 1.03, *n* = 6).

**TABLE 3:** Summary of *Nab*‐sirolimus pharmacokinetics.^a^

*Nab*‐sirolimus (mg/m^2^/dose)	All dose levels	Dose level 1 (35 mg/m^2^/dose)	Dose level – 1 (20 mg/m^2^/dose)	Dose level – 2 (15 mg/m^2^/dose)
cycle 1				
*N*	31	5	11	15
Half‐life (h)	43.8 ± 16.2	47.3 ± 9.7	47.4 ± 24.0	40.1 ± 9.8
*T*_max_ (h)	0.58 ± 0.19	0.52 ± 0.02	0.69 ± 0.28	0.52 ± 0.09
*C*_max_ (μg/mL)	—	785 ± 311	436 ± 136	464 ± 254
AUC_inf_ (μg/mL·h)	—	12,252 ± 3129	9948 ± 3824	8120 ± 2088
CL (L/h/m^2^)	2.27 ± 0.78	3.04 ± 0.78	2.29 ± 0.81	2.00 ± 0.61
Vdss (L/m^2^)	119 ± 39	163 ± 17	129 ± 43	96 ± 24
cycle 2				
*N*	15	4	3	8
Half‐life (h)	47.0 ± 11.8	47.5 ± 13.4	45.1 ± 11.6	47.5 ± 12.7
*T*_max_ (h)	0.72 ± 0.45	0.90 ± 0.48	0.51 ± 0.01	0.71 ± 0.52
*C*_max_ (ng/mL)	—	628 ± 182	506 ± 187	486 ± 261
AUC_inf_ (ng/mL*h)	—	16,369 ± 6261	7816 ± 2082	8677 ± 2524
CL (L/h/m^2^)	2.07 ± 0.77	2.43 ± 1.05	2.72 ± 0.68	1.64 ± 0.33
Vdss (L/m^2^)	109 ± 36	115 ± 28	142 ± 38	94 ± 34

^a^ Data are presented as mean with a standard deviation.

Sixteen patients provided samples for PK analysis of irinotecan. One patient was omitted from the analysis due to a discontinuation of the *Nab*‐sirolimus infusion in cycle 2. The concentration versus time curve is shown in Figure 1C and a summary of the calculated parameters is shown in Table 3. There did not appear to be a relationship between the *Nab*‐sirolimus dose level and irinotecan (Figure S1A) or SN‐38 (Figure S1B) exposure. On day 1, the mean peak irinotecan concentration (103 ng/mL) was achieved 1.48 h after oral administration and the apparent oral clearance was 261 L/h/m^2^. The mean peak plasma SN‐38 concentration (6.15 ng/mL) was achieved 2‐h after irinotecan administration.

### 3.2 Response

**FIGURE 2:**
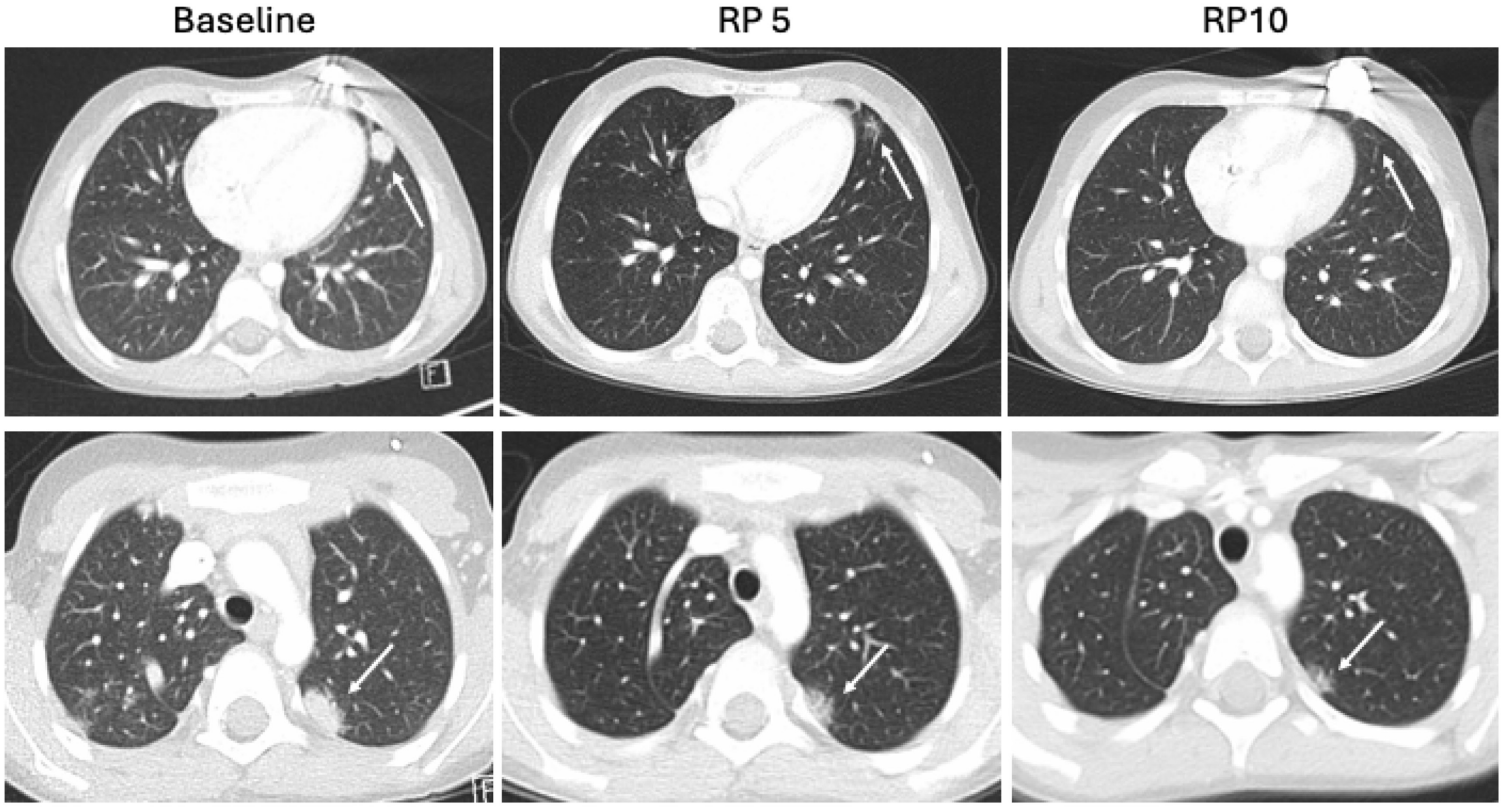
Computed tomography scan from a patient with Ewing Sarcoma treated on DL1 (*Nab*‐sirolimus [35 mg/m^2^/dose] in combination with irinotecan and temozolomide) demonstrating a confirmed partial response. RP = reporting period (cycle number).

One patient with Ewing Sarcoma treated on DL1 (*Nab*‐sirolimus 35 mg/m^2^/dose) in combination with irinotecan and temozolomide had a partial response and completed 35 cycles of protocol therapy (Figure 2). Another patient with Ewing Sarcoma who received *Nab*‐sirolimus (20 mg/m^2^/dose) had prolonged stable disease for a total of six cycles. A patient with nephroblastoma who received *Nab*‐sirolimus (15 mg/m^2^/dose) had prolonged stable disease for six cycles and withdrew from study for surgical resection per physician request. Although these results are encouraging, one must consider the role irinotecan and temozolomide played in response as this combination has previously been documented to be active in a variety of pediatric solid tumors.

## 4 Discussion

This is the first clinical trial of *Nab*‐sirolimus in combination with irinotecan and temozolomide in pediatric patients (Table 4). The primary goal was to determine the dose of *Nab*‐sirolimus that could be administered in combination with temozolomide and irinotecan and describe the PK and toxicity of *Nab*‐sirolimus alone and in combination. The MTD of *Nab*‐sirolimus is 15 mg/m^2^/dose on days 1 and 8 in combination with temozolomide (125 mg/m^2^/dose) and oral irinotecan (90 mg/m^2^/dose) daily on days 1–5. The requirement for administration of 85% of planned doses of protocol therapy and completion of toxicity evaluation for both cycle 1 and cycle 2 to determine MTD/RP2D increased the number of patients who were not fully evaluable for toxicity due to disease progression during cycle 1. Initially, patients who experienced disease progression after *Nab*‐sirolimus alone discontinued protocol therapy without receiving combination therapy. The study was amended and allowed patients with disease progression the option to receive combination therapy. By amending the study, we were able to explore the potential synergistic toxicity and activity of *Nab*‐sirolimus in combination with irinotecan and temozolomide. We demonstrated that this design can be implemented to maximize the understanding of an investigational agent alone and in combination. However, requiring completion of both cycle 1 and cycle 2 for the DLT interval to determine the MTD resulted in increase in patients who were not fully evaluable for the primary end point (toxicity).

**TABLE 4:** Summary of the pharmacokinetics of irinotecan and its metabolites following intravenous administration of 90 mg/m^2^ irinotecan.

No. of patients	15
Irinotecan	
Half‐life (h)	6.00 ± 4.38
*T*_max_ (h)	1.51 ± 0.98
*C*_max_ (ng/mL)	103 ± 90
AUC_0‐24h_ (ng/mL*h)	851 ± 799
AUC_inf_ (ng/mL*h)	932 ± 808
APC	
Half‐life (h)	9.07 ± 5.32
*T*_max_ (h)	3.49 ± 31.88
*C*_max_ (ng/mL)	40.8 ± 36.8
AUC_0‐24h_ (ng/mL*h)	500 ± 537
AUC_inf_ (ng/mL*h)	587 ± 571
SN‐38	
Half‐life (h)	5.99 ± 4.01
*T*_max_ (h)	1.97 ± 1.53
*C*_max_ (ng/mL)	6.15 ± 5.72
AUC_0‐24h_ (ng/mL*h)	33.3 ± 41.1
AUC_inf_ (ng/mL*h)	45 ± 50
SN‐38G	
Half‐life (h)	12.8 ± 12.5
*T*_max_ (h)	2.97 ± 1.83
*C*_max_ (ng/mL)	15.6 ± 10.3
AUC_0‐24h_ (ng/mL*h)	165 ± 148
AUC_inf_ (ng/mL*h)	255 ± 239

In this trial, the most common DLT was thrombocytopenia which was consistent with the adult Phase I trial evaluating *Nab*‐sirolimus alone. Dose‐limiting thrombocytopenia occurred in four patients during cycle 1 (*Nab*‐sirolimus alone) and one patient (DL1) in cycle 2 (Table 2). This indicates the contribution of temozolomide to the determination of the MTD was minimal. Baseline platelet count in patients who experienced dose‐limiting thrombocytopenia (*n* = 5, median [range] 111,000 [105,000–191,000 platelets/mm^3^]) was lower than the baseline platelet count in patients who did not experience dose‐limiting thrombocytopenia (*n* = 16, 234,000 [151,000–426,000] platelets/mm^3^), *p* < 0.001 (Wilcoxon rank sum test). Previous treatment did not appear to contribute to the development of thrombocytopenia since all patients received prior chemotherapy and > 80% received prior radiation therapy. In this small cohort of patients, rapamycin disposition does not appear to be dose proportional in the evaluated dose range. In both cycles, the changes in AUC and *C*_max_ were less than dose proportional and clearance decreased by 33% as the dose was reduced from 35 mg/m^2^ to 15 mg/m^2^. There was a 33% decrease in clearance. Interindividual variability in rapamycin systemic exposure was modest across the various dosing cohorts in cycle 1 and cycle 2 (%CV ≤ 29% and ≤ 43%, respectively). The *C*_max_, total systemic exposure (AUC_inf_), and half‐life for rapamycin were similar between the two cycles suggesting that rapamycin PK were not changed when *Nab*‐sirolimus was given in combination with irinotecan and temozolomide.

The *Nab*‐sirolimus starting dose (35 mg/m^2^) was selected based on the adult PK data for *Nab*‐sirolimus and temsirolimus, as well as the pediatric Phase I trial of temsirolimus in combination with irinotecan and temozolomide [2, 9, 11]. The mean rapamycin exposure (AUC_inf_ = 12,252 h·ng/mL) at the 35 mg/m^2^ dose level in this study was ~50% lower than the rapamycin exposure (24,564 h·ng/mL) following 45 mg/m^2^ *Nab*‐sirolimus in adults [9] and similar to the rapamycin exposure (11,973 h·ng/mL) following 35 mg/m^2^ temsirolimus in adults [11]. Consistent with the difference in exposure, rapamycin clearance in children was higher compared to adults (3.04 L/h/m^2^ vs. 1.87 L/h/m^2^). The mean rapamycin exposure at the 15 mg/m^2^ dose level (AUC_inf_ = 8120 h·ng/mL) in this study was similar to the sum of the temsirolimus plus rapamycin exposure (10,218 h·ng/mL) following 25 mg/m^2^ temsirolimus in a pediatric Phase I study of temsirolimus alone [13]. These observations illustrate the challenges in selecting equivalent doses of rapamycin analogs for clinical trials.

The oral irinotecan PK were compared to a Phase I trial of oral irinotecan (90 mg/m^2^) combined with temozolomide and vincristine [14] and other early phase clinical trials of intravenous irinotecan (50 mg/m^2^) alone [15] or in combination with alisertib [16, 17]. The peak plasma concentrations and AUC_inf_ for SN‐38 and SN‐38G found in this study were similar to the values found in prior trials [14, 15]. However, the irinotecan (CTP‐11) and APC *C*_max_ and AUC_inf_ were higher than previously reported after oral dosing [14], and lower than those found after intravenous administration of irinotecan [15]. The higher irinotecan and APC exposure in this study may be due to the inhibition of p‐glycoprotein (P‐gp) by rapamycin. Rapamycin is a substrate for CYP3A4 and an inhibitor of P‐gp [18, 19, 20]. Given that irinotecan is a substrate for P‐gp competitive inhibition of [21], irinotecan may lead to reduced excretion and increased irinotecan AUC.

We conducted this dose finding trial in children with refractory or recurrent solid tumors without biomarker selection for TORC 1 or TORC2 in tumors. A limitation of this trial was the inability to complete assessment of archival tumor tissue expression of the downstream targets, S6K and 4E‐BP1. Therefore, it is unknown if *Nab*‐sirolimus (15 mg/m^2^) provides sufficient target inhibition.

Response was not a primary objective of this clinical trial; however, 2/5 patients with Ewings Sarcoma experienced partial response or prolonged stable disease at *Nab*‐sirolimus dose levels of 35 or 20 mg/m^2^. Further evaluation of *Nab*‐sirolimus in combination with irinotecan and temozolomide may be warranted.

## 5 Conclusions

The MTD for *Nab*‐sirolimus is 15 mg/m^2^/dose days 1 and 8 in combination with five daily doses of temozolomide 125 mg/m^2^/dose and oral irinotecan 90 mg/m^2^/dose. Rapamycin showed a nonlinear increase in exposure and peak concentration over the dose range; irinotecan had no significant impact on the pharmacokinetics of rapamycin. The trial design of investigational agent monotherapy followed by combination provided the opportunity to assess single agent toxicity, PK and preliminary activity prior to combination administration and presents a new paradigm for the development of combination regimens in children with cancer.

Children's Oncology Group Data Sharing Statement: The Children's Oncology Group Data Sharing policy describes the release and use of COG individual subject data for use in research projects in accordance with National Clinical Trials Network (NCTN) Program and NCI Community Oncology Research Program (NCORP) Guidelines. Only data expressly released from the oversight of the relevant COG Data and Safety Monitoring Committee (DSMC) are available to be shared. Data sharing will ordinarily be considered only after the primary study manuscript is accepted for publication. For phase 3 studies, individual‐level de‐identified datasets that would be sufficient to reproduce results provided in a publication containing the primary study analysis can be requested from the NCTN/NCORP Data Archive at https://nctn‐data‐archive.nci.nih.gov/. Data are available to researchers who wish to analyze the data in secondary studies to enhance the public health benefit of the original work and agree to the terms and conditions of use. For non‐phase 3 studies, data are available following the primary publication. An individual‐level de‐identified dataset containing the variables analyzed in the primary results paper can be expected to be available upon request. Requests for access to COG protocol research data should be sent to: datarequest@childrensoncologygroup.org. Data are available to researchers whose proposed analysis is found by COG to be feasible and of scientific merit and who agree to the terms and conditions of use.

For all requests, no other study documents, including the protocol, will be made available and no end date exists for requests. In addition to above, release of data collected in a clinical trial conducted under a binding collaborative agreement between COG or the NCI Cancer Therapy Evaluation Program (CTEP) and a pharmaceutical/biotechnology company must comply with the data sharing terms of the binding collaborative/contractual agreement and must receive the proper approvals.

## Author Contributions

**Stuart L. Cramer:** conceptualization (equal), data curation (equal), formal analysis (equal), investigation (equal), methodology (equal), project administration (equal), supervision (equal), writing – original draft (equal), writing – review and editing (equal). **Alyssa Terry Reddy:** conceptualization (equal), data curation (equal), formal analysis (equal), writing – review and editing (equal). **Charles Gene Minard:** data curation (equal), formal analysis (equal), writing – review and editing (equal). **Stephan Voss:** writing – review and editing (equal). **Elizabeth Fox:** conceptualization (equal), data curation (equal), formal analysis (equal), funding acquisition (equal), methodology (equal), project administration (equal), supervision (equal), writing – review and editing (equal). **Xiaowei Liu:** data curation (equal), formal analysis (equal), writing – review and editing (equal). **Kristina Denic:** data curation (equal), formal analysis (equal), writing – review and editing (equal). **Joel M. Reid:** data curation (equal), formal analysis (equal), writing – review and editing (equal). **Brenda J. Weigel:** conceptualization (equal), data curation (equal), formal analysis (equal), funding acquisition (equal), methodology (equal), project administration (equal), supervision (equal), writing – review and editing (equal).

## Disclosure

The content is solely the responsibility of the authors and does not necessarily represent the official views of the National Institutes of Health. Dr. Reid serves as a consultant for Elucida Oncology Inc.

## Conflicts of Interest

The authors declare no conflicts of interest.

## Supporting information


Appendix S1.

## Data Availability

Children's Oncology Group Data Sharing Statement: The Children's Oncology Group Data Sharing policy describes the release and use of COG individual subject data for use in research projects in accordance with National Clinical Trials Network (NCTN) Program and NCI Community Oncology Research Program (NCORP) Guidelines. Only data expressly released from the oversight of the relevant COG Data and Safety Monitoring Committee (DSMC) are available to be shared. Data sharing will ordinarily be considered only after the primary study manuscript is accepted for publication. For phase 3 studies, individual‐level de‐identified datasets that would be sufficient to reproduce results provided in a publication containing the primary study analysis can be requested from the NCTN/NCORP Data Archive at https://nctn‐data‐archive.nci.nih.gov/. Data are available to researchers who wish to analyze the data in secondary studies to enhance the public health benefit of the original work and agree to the terms and conditions of use. For non‐phase 3 studies, data are available following the primary publication. An individual‐level de‐identified dataset containing the variables analyzed in the primary results paper can be expected to be available upon request. Requests for access to COG protocol research data should be sent to: datarequest@childrensoncologygroup.org. Data is available to researchers whose proposed analysis is found by COG to be feasible and of scientific merit and who agree to the terms and conditions of use. For all requests, no other study documents, including the protocol, will be made available and no end date exists for requests. In addition to the above, release of data collected in a clinical trial conducted under a binding collaborative agreement between COG or the NCI Cancer Therapy Evaluation Program (CTEP) and a pharmaceutical/biotechnology company must comply with the data sharing terms of the binding collaborative/contractual agreement and must receive the proper approvals.
