# Supplementary material for: A Phase 1 Study of ABI‐009 (Nab‐sirolimus) in Combination With Temozolomide and Irinotecan in Pediatric Patients With Recurrent or Refractory Solid Tumors, Including CNS Tumors—A Children's Oncology Group Pediatric Early Phase Clinical Trial Network Study ADVL1514
Source: Cancer Med. 2024 Nov 2;13(21):e70376. doi: 10.1002/cam4.70376 (PMC11533328; doi:10.1002/cam4.70376)
Supplement: Supplementary file 1 — Appendix S1. [file CAM4-13-e70376-s001.docx]

Supplemental Table 1: Non-Dose Limiting Toxicities with nab-Sirolimus, Irinotecan, and Temozolomide

| Table 3. Non-DLT Toxicities Summary (maximum grade) | | | | | | | | | | |  |
| --- | --- | --- | --- | --- | --- | --- | --- | --- | --- | --- | --- |
|  | Dose Level and Toxicity Grade, No. (%) | | | | | | | | | |  |
|  | Dose Level 1(N=10) | | Dose Level -1 (N=6) | | | Dose Level -2 (N=5) | | | All Dose Level (N=21) | |  |
| Toxicity Type | All | >=Grade 3 | | All | >=Grade 3 | | All | >=Grade 3 | All | >=Grade 3 |  |
| White blood cell decreased | 4 (80) | 2 (40) | | 3 (50) | 1 (17) | | 9 (90) | 2 (20) | 16 (76) | 5 (24) |  |
| Anemia | 4 (80) | 2 (40) | | 4 (67) | 3 (50) | | 7 (70) | 0 (0) | 15 (71) | 5 (24) |  |
| Lymphocyte count decreased | 4 (80) | 1 (20) | | 4 (67) | 0 (0) | | 7 (70) | 6 (60) | 15 (71) | 7 (33) |  |
| Platelet count decreased | 5 (100) | 0 (0) | | 3 (50) | 1 (17) | | 7 (70) | 2 (20) | 15 (71) | 3 (14) |  |
| Neutrophil count decreased | 4 (80) | 3 (60) | | 2 (33) | 1 (17) | | 8 (80) | 6 (60) | 14 (67) | 10 (48) |  |
| Alanine aminotransferase increased | 4 (80) | 2 (40) | | 3 (50) | 0 (0) | | 6 (60) | 1 (10) | 13 (62) | 3 (14) |  |
| Nausea | 2 (40) | 0 (0) | | 4 (67) | 0 (0) | | 7 (70) | 0 (0) | 13 (62) | 0 (0) |  |
| Cholesterol high | 3 (60) | 0 (0) | | 2 (33) | 0 (0) | | 6 (60) | 0 (0) | 11 (52) | 0 (0) |  |
| Diarrhea | 2 (40) | 0 (0) | | 3 (50) | 0 (0) | | 6 (60) | 0 (0) | 11 (52) | 0 (0) |  |
| Hypokalemia | 5 (100) | 0 (0) | | 2 (33) | 1 (17) | | 4 (40) | 0 (0) | 11 (52) | 1 (5) |  |
| Hypophosphatemia | 5 (100) | 2 (40) | | 1 (17) | 0 (0) | | 5 (50) | 0 (0) | 11 (52) | 2 (10) |  |
| Vomiting | 3 (60) | 0 (0) | | 3 (50) | 0 (0) | | 5 (50) | 0 (0) | 11 (52) | 0 (0) |  |
| Anorexia | 3 (60) | 0 (0) | | 3 (50) | 0 (0) | | 4 (40) | 0 (0) | 10 (48) | 0 (0) |  |
| Fatigue | 4 (80) | 0 (0) | | 2 (33) | 0 (0) | | 3 (30) | 0 (0) | 9 (43) | 0 (0) |  |
| Hypertriglyceridemia | 3 (60) | 0 (0) | | 1 (17) | 0 (0) | | 5 (50) | 0 (0) | 9 (43) | 0 (0) |  |
| Aspartate aminotransferase increased | 3 (60) | 1 (20) | | 1 (17) | 0 (0) | | 4 (40) | 0 (0) | 8 (38) | 1 (5) |  |
| Hypocalcemia | 4 (80) | 0 (0) | | 3 (50) | 0 (0) | | 0 (0) | 0 (0) | 7 (33) | 0 (0) |  |
| Weight loss | 4 (80) | 0 (0) | | 1 (17) | 0 (0) | | 2 (20) | 0 (0) | 7 (33) | 0 (0) |  |
| Hypoalbuminemia | 2 (40) | 0 (0) | | 2 (33) | 0 (0) | | 2 (20) | 0 (0) | 6 (29) | 0 (0) |  |
| Hyponatremia | 1 (20) | 0 (0) | | 2 (33) | 0 (0) | | 3 (30) | 0 (0) | 6 (29) | 0 (0) |  |
| Alkaline phosphatase increased | 2 (40) | 0 (0) | | 0 (0) | 0 (0) | | 3 (30) | 0 (0) | 5 (24) | 0 (0) |  |
| Hyperglycemia | 2 (40) | 0 (0) | | 0 (0) | 0 (0) | | 3 (30) | 0 (0) | 5 (24) | 0 (0) |  |
| Sinus tachycardia | 1 (20) | 0 (0) | | 1 (17) | 0 (0) | | 3 (30) | 0 (0) | 5 (24) | 0 (0) |  |
| Abdominal pain | 1 (20) | 0 (0) | | 1 (17) | 0 (0) | | 2 (20) | 0 (0) | 4 (19) | 0 (0) |  |
| Constipation | 0 (0) | 0 (0) | | 1 (17) | 0 (0) | | 3 (30) | 0 (0) | 4 (19) | 0 (0) |  |
| Mucositis oral | 2 (40) | 0 (0) | | 1 (17) | 0 (0) | | 1 (10) | 0 (0) | 4 (19) | 0 (0) |  |
| Creatinine increased | 0 (0) | 0 (0) | | 0 (0) | 0 (0) | | 3 (30) | 0 (0) | 3 (14) | 0 (0) |  |
| Headache | 0 (0) | 0 (0) | | 0 (0) | 0 (0) | | 3 (30) | 0 (0) | 3 (14) | 0 (0) |  |

| Hypermagnesemia | 3 (60) | 0 (0) | 0 (0) | 0 (0) | 0 (0) | 0 (0) | 3 (14) | 0 (0) |
| --- | --- | --- | --- | --- | --- | --- | --- | --- |
| Hypertension | 1 (20) | 0 (0) | 0 (0) | 0 (0) | 2 (20) | 0 (0) | 3 (14) | 0 (0) |
| Lipase increased | 2 (40) | 0 (0) | 0 (0) | 0 (0) | 1 (10) | 0 (0) | 3 (14) | 0 (0) |
| Rash acneiform | 1 (20) | 0 (0) | 1 (17) | 0 (0) | 1 (10) | 0 (0) | 3 (14) | 0 (0) |
| Rash maculo-papular | 1 (20) | 0 (0) | 1 (17) | 0 (0) | 1 (10) | 0 (0) | 3 (14) | 0 (0) |
| Alopecia | 0 (0) | 0 (0) | 1 (17) | 0 (0) | 1 (10) | 0 (0) | 2 (10) | 0 (0) |
| Blood bicarbonate decreased | 0 (0) | 0 (0) | 0 (0) | 0 (0) | 2 (20) | 0 (0) | 2 (10) | 0 (0) |
| Blood bilirubin increased | 0 (0) | 0 (0) | 1 (17) | 0 (0) | 1 (10) | 0 (0) | 2 (10) | 0 (0) |
| Chills | 1 (20) | 0 (0) | 1 (17) | 0 (0) | 0 (0) | 0 (0) | 2 (10) | 0 (0) |
| Cough | 1 (20) | 0 (0) | 0 (0) | 0 (0) | 1 (10) | 0 (0) | 2 (10) | 0 (0) |
| Dry skin | 0 (0) | 0 (0) | 2 (33) | 0 (0) | 0 (0) | 0 (0) | 2 (10) | 0 (0) |
| Dysgeusia | 0 (0) | 0 (0) | 0 (0) | 0 (0) | 2 (20) | 0 (0) | 2 (10) | 0 (0) |
| Epistaxis | 1 (20) | 0 (0) | 0 (0) | 0 (0) | 1 (10) | 0 (0) | 2 (10) | 0 (0) |
| Hyperphosphatemia | 1 (20) | 0 (0) | 0 (0) | 0 (0) | 1 (10) | 1 (10) | 2 (10) | 1 (5) |
| Pain in extremity | 1 (20) | 0 (0) | 1 (17) | 0 (0) | 0 (0) | 0 (0) | 2 (10) | 0 (0) |
| Serum amylase increased | 1 (20) | 0 (0) | 1 (17) | 0 (0) | 0 (0) | 0 (0) | 2 (10) | 0 (0) |
| Blood lactate dehydrogenase increased | 0 (0) | 0 (0) | 0 (0) | 0 (0) | 1 (10) | 0 (0) | 1 (5) | 0 (0) |
| Dehydration | 1 (20) | 0 (0) | 0 (0) | 0 (0) | 0 (0) | 0 (0) | 1 (5) | 0 (0) |
| Enterocolitis infectious | 1 (20) | 0 (0) | 0 (0) | 0 (0) | 0 (0) | 0 (0) | 1 (5) | 0 (0) |
| Fever | 0 (0) | 0 (0) | 0 (0) | 0 (0) | 1 (10) | 1 (10) | 1 (5) | 1 (5) |
| Folliculitis | 0 (0) | 0 (0) | 0 (0) | 0 (0) | 1 (10) | 0 (0) | 1 (5) | 0 (0) |
| Gastrointestinal disorders - Other, Lip Lesion | 0 (0) | 0 (0) | 0 (0) | 0 (0) | 1 (10) | 0 (0) | 1 (5) | 0 (0) |
| Glucosuria | 1 (20) | 0 (0) | 0 (0) | 0 (0) | 0 (0) | 0 (0) | 1 (5) | 0 (0) |
| Gum infection | 1 (20) | 1 (20) | 0 (0) | 0 (0) | 0 (0) | 0 (0) | 1 (5) | 1 (5) |
| Hematuria | 0 (0) | 0 (0) | 0 (0) | 0 (0) | 1 (10) | 0 (0) | 1 (5) | 0 (0) |
| Hemoglobinuria | 0 (0) | 0 (0) | 0 (0) | 0 (0) | 1 (10) | 0 (0) | 1 (5) | 0 (0) |
| Hypercalcemia | 0 (0) | 0 (0) | 1 (17) | 0 (0) | 0 (0) | 0 (0) | 1 (5) | 0 (0) |
| Hyperkalemia | 0 (0) | 0 (0) | 0 (0) | 0 (0) | 1 (10) | 0 (0) | 1 (5) | 0 (0) |
| Hypernatremia | 1 (20) | 0 (0) | 0 (0) | 0 (0) | 0 (0) | 0 (0) | 1 (5) | 0 (0) |
| Hypomagnesemia | 1 (20) | 0 (0) | 0 (0) | 0 (0) | 0 (0) | 0 (0) | 1 (5) | 0 (0) |
| Hypotension | 0 (0) | 0 (0) | 0 (0) | 0 (0) | 1 (10) | 0 (0) | 1 (5) | 0 (0) |
| Hypoxia | 0 (0) | 0 (0) | 0 (0) | 0 (0) | 1 (10) | 0 (0) | 1 (5) | 0 (0) |
| INR increased | 0 (0) | 0 (0) | 1 (17) | 0 (0) | 0 (0) | 0 (0) | 1 (5) | 0 (0) |
| Insomnia | 0 (0) | 0 (0) | 0 (0) | 0 (0) | 1 (10) | 0 (0) | 1 (5) | 0 (0) |
| Investigations - Other, HYPOCHLOREMIA | 0 (0) | 0 (0) | 1 (17) | 0 (0) | 0 (0) | 0 (0) | 1 (5) | 0 (0) |
| Malaise | 1 (20) | 0 (0) | 0 (0) | 0 (0) | 0 (0) | 0 (0) | 1 (5) | 0 (0) |
| Neck edema | 0 (0) | 0 (0) | 0 (0) | 0 (0) | 1 (10) | 0 (0) | 1 (5) | 0 (0) |
| Neck pain | 0 (0) | 0 (0) | 0 (0) | 0 (0) | 1 (10) | 0 (0) | 1 (5) | 0 (0) |
| Non-cardiac chest pain | 1 (20) | 0 (0) | 0 (0) | 0 (0) | 0 (0) | 0 (0) | 1 (5) | 0 (0) |
| Oral pain | 1 (20) | 0 (0) | 0 (0) | 0 (0) | 0 (0) | 0 (0) | 1 (5) | 0 (0) |
| Peripheral motor neuropathy | 0 (0) | 0 (0) | 1 (17) | 0 (0) | 0 (0) | 0 (0) | 1 (5) | 0 (0) |
| Pneumonitis | 1 (20) | 0 (0) | 0 (0) | 0 (0) | 0 (0) | 0 (0) | 1 (5) | 0 (0) |
| Proteinuria | 1 (20) | 0 (0) | 0 (0) | 0 (0) | 0 (0) | 0 (0) | 1 (5) | 0 (0) |
| Pruritus | 1 (20) | 0 (0) | 0 (0) | 0 (0) | 0 (0) | 0 (0) | 1 (5) | 0 (0) |
| Respiratory, thoracic and mediastinal disorders - Other, tachypnea | 0 (0) | 0 (0) | 0 (0) | 0 (0) | 1 (10) | 0 (0) | 1 (5) | 0 (0) |
| Rhinorrhea | 0 (0) | 0 (0) | 1 (17) | 0 (0) | 0 (0) | 0 (0) | 1 (5) | 0 (0) |
| Sinus bradycardia | 1 (20) | 0 (0) | 0 (0) | 0 (0) | 0 (0) | 0 (0) | 1 (5) | 0 (0) |
| Skin and subcutaneous tissue disorders - Other, ATOPIC DERMATITIS | 1 (20) | 0 (0) | 0 (0) | 0 (0) | 0 (0) | 0 (0) | 1 (5) | 0 (0) |
| Skin and subcutaneous tissue disorders - Other, Diaper rash | 0 (0) | 0 (0) | 0 (0) | 0 (0) | 1 (10) | 0 (0) | 1 (5) | 0 (0) |
| Skin infection | 0 (0) | 0 (0) | 0 (0) | 0 (0) | 1 (10) | 1 (10) | 1 (5) | 1 (5) |
| Somnolence | 1 (20) | 0 (0) | 0 (0) | 0 (0) | 0 (0) | 0 (0) | 1 (5) | 0 (0) |
| Sore throat | 1 (20) | 0 (0) | 0 (0) | 0 (0) | 0 (0) | 0 (0) | 1 (5) | 0 (0) |
| Urinary frequency | 0 (0) | 0 (0) | 0 (0) | 0 (0) | 1 (10) | 0 (0) | 1 (5) | 0 (0) |
| Urinary tract pain | 0 (0) | 0 (0) | 0 (0) | 0 (0) | 1 (10) | 0 (0) | 1 (5) | 0 (0) |
| Urinary urgency | 0 (0) | 0 (0) | 0 (0) | 0 (0) | 1 (10) | 0 (0) | 1 (5) | 0 (0) |


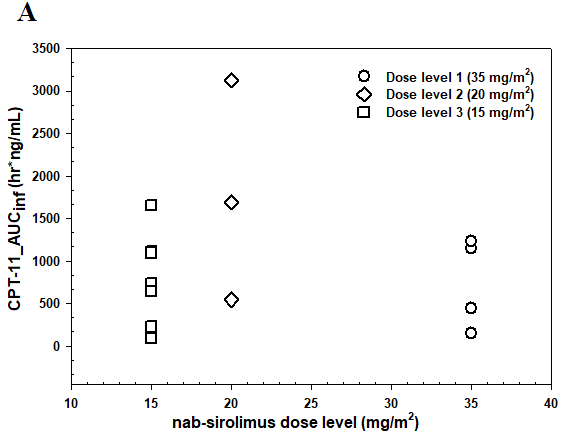


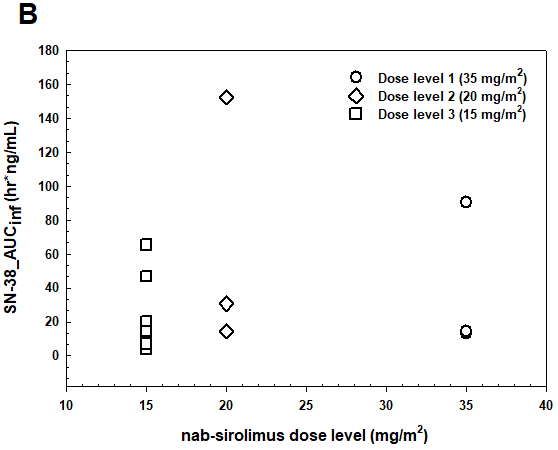


Supplemental Figure 1. Graphs of irinotecan (A) and SN-38 (B) Day 1 AUCinf *vs* nab-sirolimus dose level.


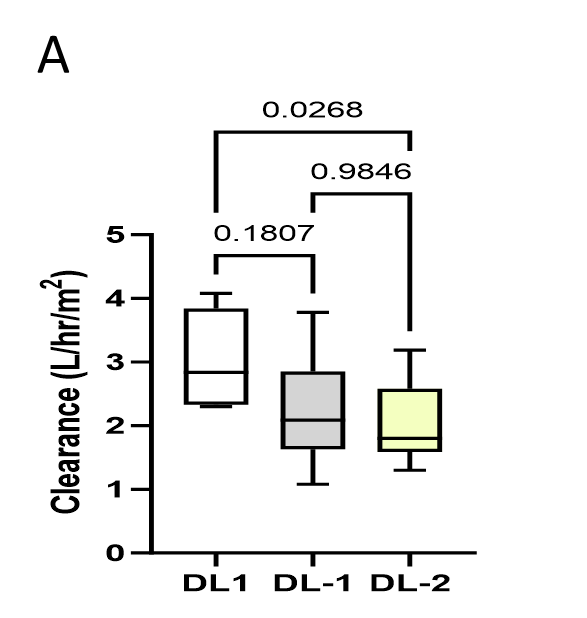


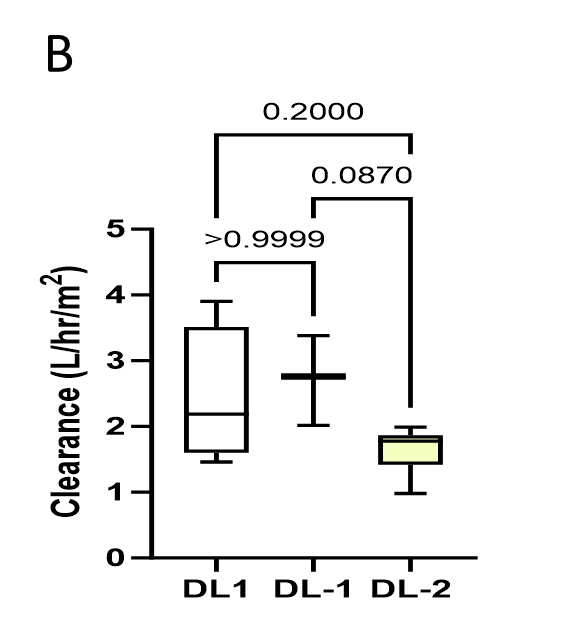


Supplemental Figure 2. Rapamycin clearance *vs* dose level in Cycle 1 (A) and Cycle 2 (B)
